# Confined conversion of CuS nanowires to CuO nanotubes by annealing-induced diffusion in nanochannels

**DOI:** 10.1186/1556-276X-6-150

**Published:** 2011-02-16

**Authors:** Cheng Mu, Junhui He

**Affiliations:** 1 Functional Nanomaterials Laboratory and Key Laboratory of Photochemical Conversion and Optoelectronic Materials, Technical Institute of Physics and Chemistry, Chinese Academy of Sciences, Zhongguancun Beiyitiao 2, Haidianqu, Beijing 100190, China

## Abstract

Copper oxide (CuO) nanotubes were successfully converted from CuS nanowires embedded in anodic aluminum oxide (AAO) template by annealing-induced diffusion in a confined tube-type space. The spreading of CuO and formation of CuO layer on the nanochannel surface of AAO, and the confinement offered by AAO nanochannels play a key role in the formation of CuO nanotubes.

## Introduction

Well-aligned semiconductor one-dimensional (1D) nanostructures have attracted extensive attention in the last decade owing to their great potential in novel optoelectronic nanodevices, such as laser diodes, field effect transistors, light-emitting diodes, and sensors [1]. Copper oxide (CuO) is a *p*-type semiconductor with a narrow band gap, and is a candidate material for photothermal and photoconductive applications [2,3]. Moreover, it is potentially a useful component in the fabrication of sensors, field emitters, lithium-CuO electrochemical cells, cathode materials, and high Tc-superconductors [4,5]. Its crystallinity, size, and shape and stoichiometry play a key role in these applications. Considerable efforts have been devoted to overcoming numerous challenges associated with efficient, controlled fabrication of these nanostructures via chemical or physical approaches. Thus far, well-aligned 1 D CuO nanostructures have been obtained using techniques such as thermal evaporation [2,6], electrospinning [7], MOCVD [8], and sol-gel process [9]. CuO nanowires were also prepared by conversion from their nanoscale analogs of copper hydroxide at elevated temperatures [10-14]. In this study, a novel approach for the preparation of CuO nanotubes via confined conversion from CuS nanowires by annealing-induced diffusion in nanochannels is reported.

Recently, prior studies including these of the authors have reported the preparation of metal sulfide nanowires by chemical precipitation in anodic aluminum oxide (AAO) channels under ambient conditions [15,16]. In this article, the authors report on the synthesis of CuO nanotubes using CuS nanowires embedded in AAO as precursor. Not only the structure but also the morphology of product could be selectively controlled via this method. The conversion too was easily performed. This approach may be extended to the synthesis of various metal oxide nanotubes by annealing their precursor nanowires embedded in AAO template, and the precursor can be sulfides, carbonates, and oxalates, which can be readily transformed into oxides at elevated temperatures.

## Experimental section

### Preparation

AAO templates used were prepared by aluminum anodic oxidation as described previously [17]. In brief, electropolished aluminum foil was anodized in aqueous oxalic acid (4%) at a constant voltage of 40 V for several hours to prepare AAO templates of 50-nm pores using a H-type cell. After the anodization, the remaining aluminum was etched by a 20% HCl + 0.2 M CuCl_2 _mixed solution, and the barrier layer was dissolved by 5% phosphoric acid.

In a typical synthesis of CuS nanowires, one half-cell of the H-type cell was filled with aqueous (NH_4_)_2_S of 0.01 M, and the other was filled with aqueous CuSO_4 _of stoichiometric concentration. After reaction for 12 h, the AAO template embedded with CuS nanowires were detached and thoroughly washed with deionized water and subsequently annealed in muffle furnace in air at 650°C for 1-20 h.

### Materials characterization

Crystallographic and purity information on as-prepared metal sulfide nanowires were obtained using powder X-ray diffraction (XRD). The XRD analyses were performed using a Philip X'Pert PRO SUPER çA rotation anode with Ni-filtered Cu Kα radiation (*λ *= 1.5418 Å). Identical slit width and accelerating voltage were used for all the samples.

CuS nanowires and CuO nanotubes were observed on a field emission scanning electron microscopy (SEM) instrument (FE-SEM Leo 1550) operated at an acceleration voltage of 10 kV. The CuS nanowires and CuO nanotubes were recovered by dissolving the AAO membrane in 2 M aqueous NaOH for 2 h at room temperature. The products were obtained by centrifugation followed by washing three times with deionized water and dried in air. Samples were dropped onto silicon wafer which was ultimately attached onto the surface of SEM specimen stage. For the analysis of nanowire arrays, membranes were initially attached to a piece of silicon wafer by conductive double-sided carbon tape. They were immersed in 0.2 M aqueous NaOH for 1 h in order to partially remove the template, creating aligned nanowires/nanotubes. After washing with deionized water followed by air-drying, the specimens were subsequently mounted onto a SEM specimen stage for imaging.

Specimens for transmission electron microscopy (TEM) and high-resolution TEM (HRTEM) observations were prepared by dropping the as-prepared nanowires/nanotubes onto carbon-coated copper grids followed by drying. TEM images and selected area electron diffraction (SAED) patterns were obtained on a JEOL JEM-2100 TEM, and HRTEM images were obtained on a JEOL JEM-2100F TEM.

## Results and discussion

**Figure 1:**
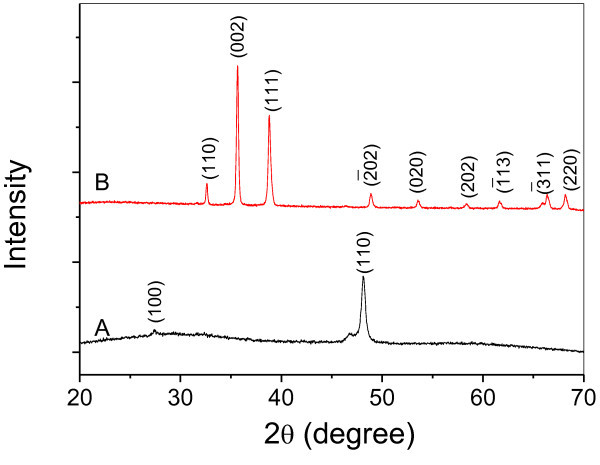
**XRD patterns of as-prepared CuS nanowires (a) and CuO nanotubes (b) using AAO template with 50-nm pores**.

The purity and crystallinity of as-prepared CuS nanowires and CuO nanotubes were characterized by XRD measurements before removing the AAO membrane. Figure 1 shows XRD patterns collected in the 2-theta range of 20-70° for the samples of both CuS nanowire and CuO nanotube. All the peaks in Figure 1a could be ascribed to hexagonal CuS (cell constants *a *= 3.796 Å, *c *= 16.38 Å; JCPDS Card No. 78-0876). The only strong XRD peak in Figure 1a indicates that the CuS nanowires have preferred (110) orientation, and all the peaks in Figure 1b could be readily indexed as monoclinic CuO (cell constants *a *= 4.6 Å, *b *= 3.4 Å, *c *= 5.1 Å; JCPDS Card No. 80-1917).

**Figure 2:**
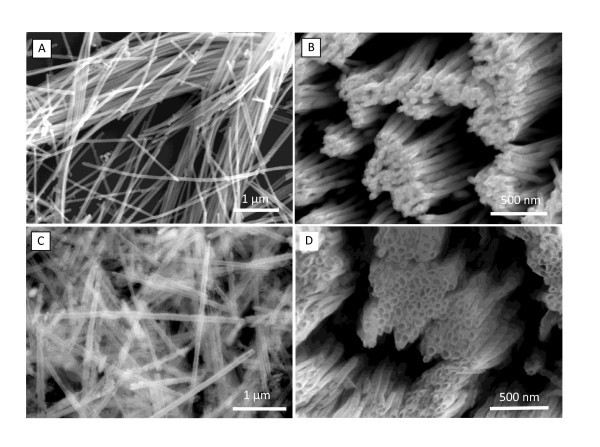
**Typical SEM images of CuS nanowires**. **(a)**; array **(b)**; CuO nanotubes **(c)**; and array **(d) **fabricated using AAO template with 50-nm pores.

The size and morphology of the as-synthesized CuS nanowire and CuO nanotube were examined by SEM. Figure 2 shows SEM images of the as-prepared CuS nanowires and CuO nanotubes. Figure 2a is a typical SEM of CuS nanowires which were prepared using an AAO template with a pore size as small as 50 nm. The nanowires are straight, and uniform in size along their axial direction. Their diameters are in the range of 50 ± 5 nm, which agree well with those of the pores of the AAO template used, indicating fine confinement of the template pores. Figure 2b gives a SEM top view of the CuS nanowire array after partly dissolving the AAO pore wall. The nanowires tend to "stick" to each other due to capillary force. Figure 2c is a typical SEM image of CuO nanotubes. It presents a large number of nanotubes without any visible byproducts, suggesting that the product is of high purity. The nanotube diameter ranges from 50 to 60 nm. Their surfaces are not quite smooth. Figure 2d shows a top view of CuO nanotube array, clearly showing the open-ends of the nanotubes.

**Figure 3:**
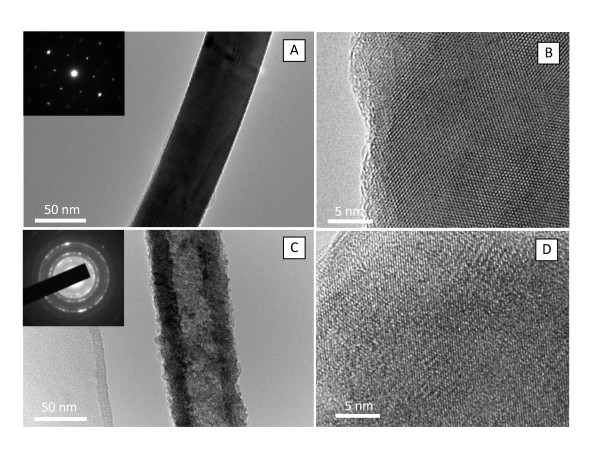
**TEM images of a single CuS nanowire**. **(a) **and CuO nanotube **(c) **with a diameter of 50 nm. The insets in **(a, c) **are the electron diffraction patterns of the CuS nanowire and CuO nanotube. HRTEM images of CuS nanowire **(b)**, and CuO nanotube **(d)**.

The morphology of the CuO nanotubes was further confirmed by TEM observations. Figure 3a is a typical TEM image of the CuS nanowire, indicating that the nanowire possesses a smooth surface and a uniform diameter of ca. 50 nm that is again in good agreement with that of the AAO pore. The inset of Figure 3a shows the SAED spots of CuS nanowire, and could be well assigned to the hexagonal crystal system, in agreement with the above XRD results. The clear distribution of spots indicates the single crystal nature of the CuS nanowire. The HRTEM image of CuS nanowire (Figure 3b) with clearly visible lattice fringes also provides the evidence of single-crystal nature. A typical TEM image of the CuO nanotube is shown in Figure 3c. The inner/outer surfaces of the CuO nanotube were not quite smooth as compared to the CuS nanowire, and its diameter was estimated to be ca. 55 nm, which is larger than that of the CuS nanowire. The SAED analysis on the CuO nanotube gave a clear electron diffraction pattern (the inset of Figure 3c) composed of several rings. At least three diffraction rings could be identified, with average *d *spacings of 2.53 and 2.52 Å associated with the 002 and -111 reflections, 2.32 and 2.31 Å associated with the 111 and 200 reflections, and 1.87 Å associated with the -202 reflection. The SAED results, in accordance with the XRD data, demonstrate that the CuO nanotube is polycrystalline of the monoclinic phase, and has lost the preferred orientation. The HRTEM image of CuO nanotube shown in Figure 3d further identifies a polycrystalline structure.

**Figure 4:**
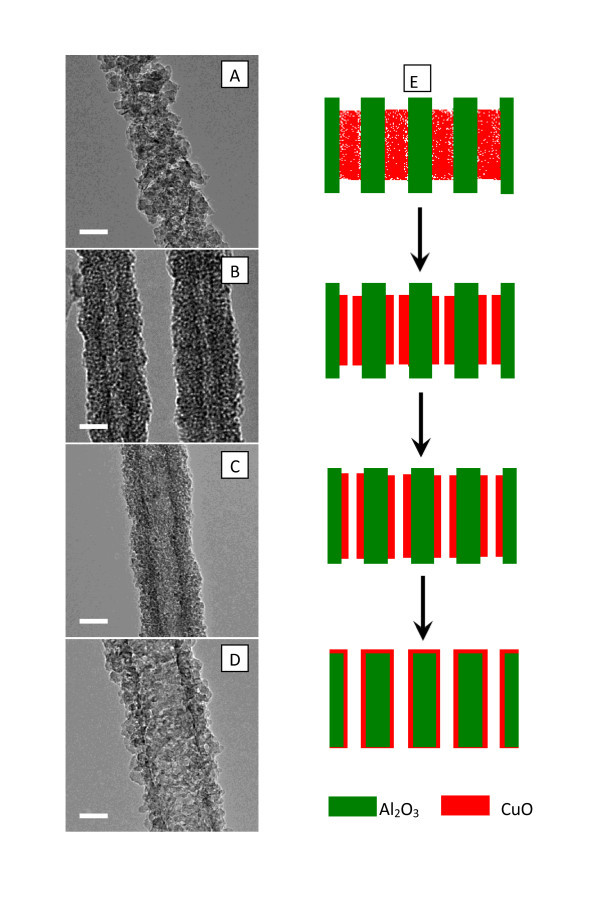
**TEM images of CuO nanowires and nanotubes obtained by annealing at 650°C for varying periods of time**: **(a) **1 h, **(b) **4 h, **(c) **10 h, and **(d) **20 h. The scale bars in **(a-d) **are 20 nm. **(e) **Schematic illustration of the growth process of CuO nanotubes.

A hypothesis for the formation mechanism of CuO nanotubes from CuS nanowires was that, at elevated temperature, CuO was formed by oxidation of CuS, and might be spread on the pore surface of AAO template. It was previously reported that CuO could form a monolayer spontaneously on the Al_2_O_3 _surface at a temperature much lower than its melting point [18,19]. Once a CuO layer is formed on the pore surface of AAO template, further spreading of CuO would become possible, which would eventually result in the formation of CuO nanotubes. To examine this hypothesis for the formation mechanism of CuO nanotubes, CuS nanowires embedded in AAO template were annealed in muffle furnace at 650°C for varying periods of time. Figure 4a,b,c,d shows TEM images of CuO nanostructures obtained by annealing CuS nanowires embedded in AAO for 1, 4, 10, and 20 h, respectively. After 1-h annealing, the CuS nanowires of smooth surface were converted to CuO nanowires of rough surface, which consist of small aggregated CuO particles. This is in sharp contrast to the single crystal structure of precursor CuS nanowires. After annealing for 4-20 h, the CuS nanowires turned to tube-type CuO nanostructures. The wall thickness of tube-type CuO nanostructure became thinner with increase of annealing time, and for extended annealing (e.g., 20 h), the exterior surface of AAO template was found to be covered by a thin CuO layer. This clearly indicated that CuO had spread on the channel surface and exterior surface of AAO template. Figure 4e schematically illustrates the process of CuO nanotube growth. In contrast, nanowires without the support of AAO template would break under different heat-treatment conditions, leading to the formation of nanoparticles instead of nanotubes [20,21]. Thus, the spreading of CuO and formation of CuO layer on the nanochannel surface of AAO and the confinement offered by AAO nanochannels play a key role in the formation of CuO nanotubes. While the surface CuO layer acts as a nucleation center, the AAO nanochannels help the CuO nanowires maintain their 1 D morphology at elevated temperatures.

## Conclusions

In summary, CuO nanotubes were successfully converted from CuS nanowires embedded in AAO template by annealing-induced diffusion in a confined tube-type space. The spreading of CuO and formation of CuO layer on the nanochannel surface of AAO and the confinement offered by AAO nanochannels play a key role in the formation of CuO nanotubes. Preliminary results showed that the present conversion by annealing-induced confined diffusion of sulfide nanowires to oxide nanotubes might be readily extended to other precursors that can thermally decompose to form corresponding oxides, including carbonates and oxalates, and thus opening up a new viable route to prepare nanotubes of various oxides. Since the CuO nanotubes grew with the assistance of AAO template, their diameter and pore size could be feasibly tuned by changing the electrochemical parameters used during the fabrication of the AAO template. It is expected that such CuO nanotubes may offer exciting opportunities for applications in catalysis, electrochemistry, superconductivity, and super-hydrophobic coating. Furthermore, CuO nanotubes with large specific surface areas may also be applied in sensor applications.

## Abbreviations

AAO: anodic aluminum oxide; CuO: copper oxide; HRTEM: high-resolution TEM; SAED: selected area electron diffraction; SEM: scanning electron microscopy; TEM: transmission electron microscopy; XRD: X-ray diffraction.

## Competing interests

The authors declare that they have no competing interests.

## Authors' contributions

CM designed the experiments, carried out the sample preparation, performed SEM, TEM, HRTEM and XRD measurements and drafted the manuscript. JH coordinated the research fund and activity and helped design the experiments. Both authors took part in the discussion of the results and helped shape the final manuscript. All authors read and approved the final manuscript.

## References

[B1] Xia YN Yang PD Sun YG Wu YY Mayers B Gates B Yin YD Kim F Yan HQ "One-dimensional nanostructures: Synthesis, characterization, and applications" Adv Mater 2003 15 353 10.1002/adma.200390087

[B2] Jiang XC Herricks T Xia YN " CuO nanowires can be synthesized by heating copper substrates in air" Nano Lett 2002 2 1333 10.1021/nl0257519

[B3] Musa AO Akomolafe T Carter MJ " Production of cuprous oxide, a solar cell material, by thermal oxidation and a study of its physical and electrical properties" J Sol Energy Mater Sol Cells 1998 51 305 10.1016/S0927-0248(97)00233-X

[B4] Lanza F Feduzi R Fuger J "Effects of lithium oxide on the electrical properties of CuO at low temperatures" J Mater Res 1990 5 1739 10.1557/JMR.1990.1739

[B5] Podhajecky P Zabransky Z Novak P Dobiasova Z Eerny R Valvoda V "Relation between Crystallographic Microstructure and Electrochemical Properties of CuO for Lithium Cells" ElectrochimActa 1990 35 245 10.1016/0013-4686(90)85065-U

[B6] Cheng CL Ma YR Chou MH Huang CY Yeh V Wu SY "Direct observation of short-circuit diffusion during the formation of a single cupric oxide nanowire" Nanotechnology 2007 18 245604 10.1088/0957-4484/18/24/245604

[B7] Wu H Lin DD Pan W "Fabrication, assembly, and electrical characterization of CuO nanofibers" Appl Phys Lett 2006 89 133125 10.1063/1.2355474

[B8] Malandrino G Finocchiaro ST Nigro RL Bongiorno C Spinella C Fragalà IL "Free-standing copper(II) oxide nanotube arrays through an MOCVD template process" Chem Mater 2004 16 5559 10.1021/cm048685f

[B9] Su YK Shen CM Yang HT Li HL Gao HJ "Controlled synthesis of highly ordered CuO nanowire arrays by template-based sol-gel route" Trans Nonferrous Met Soc China 2007 17 783 10.1016/S1003-6326(07)60174-5

[B10] Cao MH Hu CW Wang YH Guo Y Guo CX Wang EB "A controllable synthetic route to Cu, Cu2O, and CuO nanotubes and nanorods" Chem Commun 2003 1884 10.1039/b304505f 12932015

[B11] Du GH Tendeloo GV "Cu(OH)2 nanowires, CuO nanowires and CuO nanobelts" Chem Phys Lett 2004 393 64 10.1016/j.cplett.2004.06.017

[B12] Lu CH Qi LM Yang JH Zhang DY Wu NZ Ma JM "Simple template-free solution route for the controlled synthesis of Cu(OH)(2) and CuO nanostructures" J Phys Chem B 2004 108 17825 10.1021/jp046772p

[B13] Wen XG Xie YT Choi CL Wan KC Li XY Yang SH "Copper-based nanowire materials: Templated syntheses, characterizations, and applications" Langmuir 2005 21 4729 10.1021/la050038v 16032897

[B14] Zhang WX Ding SX Yang ZH Liu AP Qian YT Tang SP Yang SH "Growth of novel nanostructured copper oxide (CuO) films on copper foil" J Cryst Growth 2006 291 479 10.1016/j.jcrysgro.2006.03.015

[B15] Mu C He JH "Synthesis of Single Crystal Metal Sulfide Nanowires and Nanowire Arrays by Chemical Precipitation in Templates" J Nanosci Nanotechnol 2010 10 8191 10.1166/jnn.2010.2655 21121315

[B16] Zhang F Wong SS "Controlled Synthesis of Semiconducting Metal Sulfide Nanowires" Chem Mater 2009 21 4541 10.1021/cm901492f

[B17] Mu C Yu YX Wang RM Wu K Xu DS Guo GL "Uniform metal nanotube arrays by multistep template replication and electrodeposition" Adv Mater 2004 16 1550 10.1002/adma.200400129

[B18] Xie YC Tang YQ "Spontaneous monolayer dispersion of oxides and salts onto surfaces of dupports: Applications to heterogeneous catalysis" Adv Catal 1990 37 1 full_text

[B19] Wang F Wang Y Yu JF Xie YC Li JL Wu K "Template-assisted preparations of crystalline Mo and Cu nanonets" J Phys Chem C 2008 112 13121 10.1021/jp802716s

[B20] Wang SH Huang QJ Wen XG Li XY Yang SH "Thermal oxidation of Cu2 S nanowires: A template method for the fabrication of mesoscopic CuxO(x = 1,2) wires" Phys Chem Chem Phys 2002 4 3425 10.1039/b201561g

[B21] Ahmad T Ramanujachary KV Lofland SE Ganguli AK "Nanorods of manganese oxalate: a single source precursor to different manganese oxide nanoparticles (MnO, Mn2O3, Mn3O4)" J Mater Chem 2004 14 3406 10.1039/b409010a

